# Implicit Associations With Nature and Urban Environments: Effects of Lower-Level Processed Image Properties

**DOI:** 10.3389/fpsyg.2021.591403

**Published:** 2021-05-20

**Authors:** Claudia Menzel, Gerhard Reese

**Affiliations:** Social, Environmental, and Economic Psychology, University of Koblenz-Landau, Landau, Germany

**Keywords:** restoration, image properties, perceptual fluency, implicit association test, nature scenes, urban scenes

## Abstract

Nature experiences usually lead to restorative effects, such as positive affective states and reduced stress. Even watching nature compared to urban images, which are known to differ in several image properties that are processed at early stages, can lead to such effects. One potential pathway explaining how the visual input alone evokes restoration is that image properties processed at early stages in the visual system evoke positive associations. To study these automatic bottom-up processes and the role of lower-level visual processing involved in the restoring effects of nature, we conducted two studies. First, we analyzed nature and urban stimuli for a comprehensive set of image properties. Second, we investigated implicit associations in a dichotomous set of nature and urban images in three domains, namely, valence, mood, and stress restoration. To examine the role of lower-level processing in these associations, we also used stimuli that lacked the spatial information but retained certain image properties of the original photographs (i.e., phase-scrambled images). While original nature images were associated with “good,” “positive mood,” and “restoration,” urban images were associated with “bad” and “stress.” The results also showed that image properties differ between our nature and urban images and that they contribute to the implicit associations with valence, although spatial information and therefore recognition of the environment remained necessary for positive associations. Moreover, lower-level processed image properties seem to play no or only minor roles for associations with mood and stress restoration.

## Introduction

Generally, natural compared to urban environments are preferred by humans and are associated with more positive valence (e.g., Kaplan, 1987; Berto, 2007; Sánchez et al., 2016; but see also Meidenbauer et al., 2019). Many studies showed that a stay in nature, compared to a stay in an urban area, leads to improved measures of restoration (for recent reviews see Bowler et al., 2010; Bratman et al., 2012; Kuo, 2015; Corazon et al., 2019). Here, we define restoration broadly as a process in which depleted resources are renewed; it therefore includes, for example, recovering certain states of mood, attention, and physical condition (cf., Hartig, 2004, 2017). Merely watching images of nature compared to watching images of urban environments can already elicit restoration effects on certain measures of attention, mood, and parasympathetic activity (Hartig et al., 1996; Berto, 2005; Berman et al., 2008; Gamble et al., 2014; van den Berg et al., 2015). This finding suggests that isolated visual input is sufficient to evoke the reported effects. This leads to our primary research question: How does *viewing* nature affect restoration outcomes? To address this question, we investigated automatic associations with nature and urban images as well as the role of early visual processing for such associations. To introduce our research, we present relevant theories in the field of restoration evoked by nature, characteristics of nature and urban images, the processing of such properties, and their relation to naturalness, affective evaluations, and restoration.

### Theories Explaining Restoration Effects

Attention restoration theory (ART; Kaplan, 1995) and stress recovery theory (SRT; Ulrich, 1983; Ulrich et al., 1991) possibly represent the two most influential theories in the field of restorative environments. ART highlights the importance of cognitive processes and claims that so-called directed attention depletes in urban environments and restores in nature (Kaplan, 1995). It assumes that elements in nature and other restorative environments, such as mountains, lakes, or moving leaves, promote restoration by evoking feelings or states of being away and so-called soft fascination and by providing certain levels of extent and compatibility (Kaplan, 1995). ART was tested frequently and several studies showed that people who stayed in real nature or viewed virtual counterparts (compared to urban environments) performed better in some cognitive tasks (e.g., digit span tasks, which measure working memory), but not in others (e.g., visual attention tasks; for meta-analyses, see Ohly et al., 2016; Stevenson et al., 2018).

SRT claims that natural environments aid in recovering from stress via positive affective states (Ulrich, 1983; Ulrich et al., 1991). Ulrich proposed that the positive affective states and preferences may be evoked because nature comprises certain structural and visual properties (e.g., complexity, depth), a lack of threat, and/or the occurrence of water (Ulrich, 1983). In line with SRT, several studies showed that natural compared to urban environments evoke positive affective responses and reduced stress (e.g., Valtchanov and Ellard, 2010; Gladwell et al., 2012; Brown et al., 2013). Relatedly, natural environments were found to be associated with positive valence, in both explicit ratings and implicit tasks (e.g., Hietanen et al., 2007; van der Ha, 2011; Sánchez et al., 2016; Mullin et al., 2017; Schertz et al., 2018; Beute and de Kort, 2019).

Joye and van den Berg (2011) challenged the SRT by proposing a more parsimonious explanation for the beneficial effects of nature found in the literature: the perceptual fluency account (PFA). In general, perceptual fluency refers to the ease of processing of a stimulus, and the positive affect caused by this ease is assigned to the stimulus, which then leads to a positive evaluation of it (Reber et al., 2004). For example, a high-contrast image may be liked more than a similar low-contrast image, because higher contrast leads to more fluent processing and better recognizability of the depicted objects. In fact, in the field of aesthetic research, fluency—and its possibly underlying neural principle, efficient coding—is often discussed to be a key mechanism for visual preferences (Graham and Field, 2007a, b; Redies, 2007; Graham and Redies, 2010; Palmer et al., 2013). The PFA postulates that people perceive nature as more positive and restorative compared to urban environments because its processing is more fluent (Joye and van den Berg, 2011). In line with the PFA, nature images were not only rated as more aesthetic and restorative than urban images; eye tracking and blink rate data indicated less effort for viewing nature compared to urban images (Berto et al., 2008; Valtchanov and Ellard, 2015). Similarly, electroencephalogram and brain imaging data indicated that nature compared to urban images evoked fewer or less demanding processes that are assumed to be related to early visual, attentional, and/or memory processes (Tang et al., 2017; Grassini et al., 2019). Similarly, when considering only urban environments, viewing buildings with more natural compared to unnatural properties was associated with less energy in visual brain areas (Le et al., 2017). As discussed in this latter study and further work including those on the PFA (Joye and van den Berg, 2011), these diverging processing demands may be caused by different properties of nature and urban images. We introduce such properties and their relevance for preference and restorativeness in the following section.

### Visual Properties of Nature and Urban Scenes

In comparison to random images (for example, imagine a QR code), natural images share several properties (e.g., edges, homogenous areas; Olshausen and Field, 2000; Geisler, 2008; Graham and Field, 2009). They comprise homogenous areas that lead to a correlation of brightness values for their pixels (i.e., neighboring pixels are more similar than distant pixels). This correlation leads to redundant and therefore predictable information which is processed efficiently and can be represented by a linear relation of amplitude and spatial frequencies (e.g., Graham and Field, 2009). This linear relationship is described with the so-called spectral slope (or Fourier slope or 1/*f*^α^ characteristics). It represents statistical scale invariance or regularity, which means that the characteristics of the image are relatively similar when zooming in and out of a scene. Likely because the human visual system is evolutionary (and partly ontogenetically) adapted to natural environments, images having respective properties, such as a specific spectral slope, are generally preferred and also found in aesthetic stimuli such as art, while deviating stimuli can lead to feelings of discomfort in the viewer (Redies, 2007; Graham and Field, 2009; Graham and Redies, 2010; Juricevic et al., 2010; Le et al., 2017). The spectral slope and derived measures are commonly used in visual perception research and empirical aesthetics to assess the “naturalness” of a given image. Thereby, it is generally found that spectral slopes similar to those found for natural scenes are associated with aesthetic perception and processing benefits, even across image types (e.g., Graham and Field, 2007b; Redies et al., 2007; Graham and Redies, 2010; Juricevic et al., 2010; Menzel et al., 2015, 2017; Penacchio and Wilkins, 2015; Spehar et al., 2015; Le et al., 2017).

In comparison to nature images, depictions of urban environments differ in the spectral slope and several other properties (for descriptions of the properties, see Table 1; Burton and Moorhead, 1987; Tolhurst et al., 1992; Ruderman and Bialek, 1994; Redies et al., 2007; Braun et al., 2013; Wang and Ogawa, 2015). Furthermore, edge and color properties are associated with naturalness that, by definition, differs between nature and urban environments (Table 1; Berman et al., 2014; Kardan et al., 2015; Ibarra et al., 2017). For example, non-straight edge density even affects thought content, as was suggested by a study series investigating lower-level processed characteristics of green parks and journal entries of park visitors and online study participants (Schertz et al., 2018). Many of the properties that differ between nature and urban images or vary with naturalness are processed early in the visual system and are therefore called lower-level image properties. Among these are color, brightness, contrast, and edge and spatial frequency properties.

**TABLE 1:** Overview of measured image properties, their definition, and relevance for the current work.

**Image property**	**Definition**	**Relevance**
Spectral slope	Slope of the curve of the radially averaged spatial frequency and spectral power (i.e., amplitude squared) of an image in a log–log plot	Shallower slope for nature compared to urban images (e.g., Braun et al., 2013); values similar to those of natural scenes (i.e., ≈−2.2) are associated with efficient coding, aesthetic perception, and visual discomfort (e.g., Redies, 2007; Graham and Field, 2009; Juricevic et al., 2010; Spehar et al., 2016)
Weighted residuals	Deviation of the amplitude spectrum compared to a modeled 2D amplitude spectrum with a slope of −2 in the 1D power spectrum, weighted by spatial frequency sensitivity, and adjusted by the differing energy in horizontal and vertical orientations	Deviation (higher residuals) of natural scene characteristics are associated with visual discomfort (Penacchio and Wilkins, 2015)
HSF	Sum of the power of high (> 24 cpi) spatial frequencies divided by power of all frequencies	Indirect indication for positive association with pleasure (Valtchanov and Ellard, 2015)
MSF	Sum of the power of medium (8–24 cpi) spatial frequencies divided by of all frequencies	Indirect indication for positive association with pleasure (Valtchanov and Ellard, 2015)
LSF	Sum of the power of low (< 8 cpi) spatial frequencies divided by of all frequencies	Indirect indication for positive association with cognitive load (Valtchanov and Ellard, 2015)
Self-similarity	Similarity of gradient histograms of sub-images and the entire image	Higher for nature than urban images (e.g., Braun et al., 2013); negatively associated with aesthetic evaluation in artworks (e.g., Hayn-Leichsenring et al., 2017); no association with valence in affective pictures (Redies et al., 2020)
Complexity	Sum of gradient strengths	Buildings and facades more complex than natural scenes (e.g., Braun et al., 2013); negatively associated with aesthetic evaluation in artworks (e.g., Hayn-Leichsenring et al., 2017)
Anisotropy	Distribution of gradient orientations	Lower for nature than urban images (e.g., Braun et al., 2013); positively associated with aesthetic evaluation (e.g., Hayn-Leichsenring et al., 2017)
Hue	Average dimension of color	Negatively associated with naturalness and preference (e.g., Palmer and Schloss, 2010; Ibarra et al., 2017)
Saturation	Average saturation	Positively associated with naturalness and preference (e.g., Ibarra et al., 2017)
Brightness	Average value of color	Positively associated with affective ratings (Lakens et al., 2013), naturalness and preference (e.g., Ibarra et al., 2017)
SD of hue	Standard deviation of hue across all pixels; reflects diversity of color	Negatively associated with naturalness (Berman et al., 2014; Kardan et al., 2015)
SD of saturation	Standard deviation of saturation across all pixels	Positively associated with naturalness and preference (e.g., Kardan et al., 2015)
SD of brightness	Standard deviation of all pixel values; similar to the contrast of an image	Positively associated with fluent processing (Reber et al., 2004), naturalness (e.g., Ibarra et al., 2017)
Entropy	Uniformity of intensity histogram; similar to the randomness of an image	Negatively associated with naturalness (Ibarra et al., 2017)
Edge density	Number of pixels on edges divided by the total number of pixels	Positively associated with naturalness (e.g., Kardan et al., 2015)
Straight edge density	Number of pixels on straight edges divided by the total number of pixels	Negatively associated with naturalness and preference (e.g., Berman et al., 2014; Kardan et al., 2015)
Non-straight edge density	Number of pixels on non-straight edges divided by the total number of pixels	Positively associated with naturalness (e.g., Berman et al., 2014)
Fractal dimension	Complexity measured in binary image versions by the Boxcount method	Different between nature images and certain types of urban images (e.g., Braun et al., 2013); medium values are associated with preference (e.g., Hagerhall et al., 2004)

*For further information, on the calculation of these properties, see Supplementary Table 4 of the Supplementary Material.*

The authors of the PFA argued that internal repetition—or fractal characteristics—in nature compared to other stimuli is the basis for more fluent processing (Joye and van den Berg, 2011): for example, visualize a branch and note that it looks quite similar to a whole tree. Such regularity and statistical counterparts can be measured in an image by assessing the spectral slope and other measures of self-similarity (Table 1). The spectral slope is most appropriate to consider, because of its associations with efficient coding and aesthetic processing (e.g., Graham and Field, 2007a; Redies, 2007; Juricevic et al., 2010; and also above). Worthy of note is that the fractal dimension is another measure often used to describe fractal structures, as well as natural and built scenes (e.g., Hagerhall et al., 2004; Patuano, 2018; Coburn et al., 2019), although it measures complexity rather than internal repetition. Some studies already investigated the effects of fractal dimension on restoration measures. While some of them indicated a preference and distinct brain activity for medium fractal dimensions, other studies found no clear relationship to rated restorativeness (Hagerhall et al., 2004, 2008; Hagerhall and Berto, 2008). Relatedly, Taylor and colleagues claimed that exposure to images with medium fractal dimensions leads to beneficial physiological states (Taylor et al., 2005; Taylor, 2006), but both the stimulus set and methodical approach in their study were limited and thus do not allow a valid conclusion.

**FIGURE 1:**
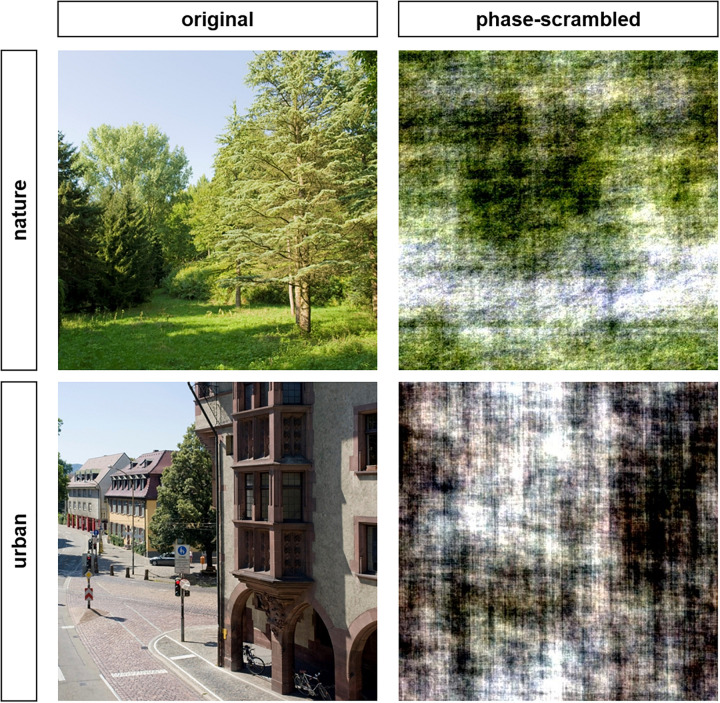
Examples of stimuli used in Studies 1 and 2. Original images were from colourbox.de and cropped to a square format.

When considering fast evaluations of environments (including nature and urban scenes), previous experiments indicated that humans assess automatic preference for their environments and that this preference is partly based on lower-level properties (including self-similarity; Mullin et al., 2017). Relatedly, Valtchanov and Ellard (2015) investigated the role of lower-level processed image properties on the perception and evaluation of nature and urban scenes. Among other image manipulations, they used so-called phase-scrambled images. In such images, the amplitude spectrum that corresponds to the distribution of spatial frequency and their amplitude is kept intact while the phase spectrum that represents the spatial information is randomized (for examples, see Figure 1; note that Valtchanov and Ellard, 2015, used a slightly different procedure to create such images and focused on gray-scaled images). In phase-scrambled images, the spectral slope, contrast, brightness (and color), and orientation properties are similar to original images, while the spatial information, and therefore object recognition, is impeded. Thus, with such images, one can study the perception of these image properties independent of recognizable content. Valtchanov and Ellard (2015) observed that participants preferred nature over urban images for both their original and phase-scrambled version (note that they used only four nature and four urban images). Thus, although this study used gray-scaled stimuli, they found a preference difference between nature and urban phase-scrambled images, indicating that spatial frequency properties, such as the spectral slope, influenced preference, independent of content and color. By using other scrambling techniques, Kotabe et al. (2016) showed that naturalness is partly coded by color but not by edge characteristics when object recognition is impeded. In sum, these studies showed that certain image properties are associated with preference and naturalness even though object recognition is not possible, and the ratings are therefore not based on evaluations of depicted objects or associations therewith. The reviewed findings support the assumption that lower-level visual processing plays a role in evoking positive affect and perhaps other restorative effects when viewing nature compared to urban images.

In sum, both computational and psychological studies show that lower-level processing plays a role in processing and evaluation of nature and urban scenes. These findings support the notion of PFA that visual characteristics (esp. regularities and color) contribute to the beneficial effects evoked by visual nature (compared to urban) experience. Here, we wanted to test the role of lower-level image properties on automatic responses to nature and urban scenes.

### Current Research

Our primary goal was to understand how *viewing* nature compared to urban images leads to restoration effects. One potential pathway is that image properties that are typical for nature evoke positive associations. In fact, PFA suggests an automatic positive evaluation of nature scenes, because of the more fluent processing of their characteristics (i.e., regularity; Joye and van den Berg, 2011). The studies reviewed above highlighted an influence of regularity measures and other lower-level processed properties on both (automatic) evaluation of and restoration evoked by environmental stimuli (e.g., Joye and van den Berg, 2011; Kardan et al., 2015; Valtchanov and Ellard, 2015; Mullin et al., 2017; Schertz et al., 2018). Therefore, we focused on studying the influence of such properties when automatically assessing nature and urban images. To test for an effect of image properties independent of the depicted environment, we used a set of nature and urban images in two versions: original and phase-scrambled.

To study the automatic evaluation of nature and urban images and their phase-scrambled versions, we focused on implicit measures. Previous studies already used such measures, for example implicit association tests (IAT), priming, or misattribution tasks, to investigate associations with nature and urban scenes (Hietanen and Korpela, 2004; Hietanen et al., 2007; Joye et al., 2013; Sánchez et al., 2016; Mullin et al., 2017). These studies generally revealed positive associations with nature. In such tests, automatic evaluations of stimuli are assessed indirectly, by measuring reaction times (IAT, priming) and/or evaluations of subsequently presented neutral stimuli (misattribution task, priming). By doing so, implicit measures have the advantage of measuring automatic evaluations which are less biased than those of explicit measurements (De Houwer et al., 2009; Greenwald et al., 2009). Furthermore, implicit measures circumvent the need of participants consciously and explicitly attributing the stimuli to certain evaluative dimensions, which is especially important for the evaluation of phase-scrambled images. Thus, by using an implicit measure, we were not only able to investigate the automatic and spontaneous reactions to phase-scrambled images but also reduced the likelihood of biased responses for the original images (e.g., social desirability, predictable aim of the experiment).

While the cited studies that used implicit measures focused on valence effects, they did not address other dimensions that are also relevant for beneficial effects of viewing nature compared to urban images. Evoked mood and affective states are important factors, especially in SRT and PFA, but also ART. Moreover, (perceived) restoration and stress were the superior outcomes studied in the field of SRT and ART, but also PFA. Consequently, affective states and restoration/stress outcomes were often-measured variables in many studies investigating the beneficial effects of nature (e.g., McMahan and Estes, 2015; Menardo et al., 2019). Therefore, they are considered in the current study, and we tested whether humans have automatic associations between nature and good, positive mood, and general restoration, and between urban scenes and bad, negative mood, and stress. Our aim to test not only one but three relevant evaluative dimensions (i.e., valence, mood, stress restoration) led to the decision for the IAT as the best suited implicit measure.

We focused on a dichotomous categorization of environments (i.e., nature vs. urban), although we are aware of its shortcomings (cf., Staats et al., 2016; Weber and Trojan, 2018). We decided to do so, because we were interested in studying the underlying mechanisms of effects evoked by such dichotomous stimulus sets or similar *in situ* environments (e.g., Berman et al., 2008; Valtchanov and Ellard, 2015; Stevenson et al., 2018). Thus, here nature was represented by diverse natural green and blue environments and included both wilderness and human-shaped German natural sceneries. The urban category represented relatively diverse scenes of German cities, including housing blocks, streets, and inner city scenes but excluded urban green parks.

We analyzed our nature and urban photographs for a comprehensive set of image properties, which were associated with affective evaluations, naturalness, and/or restoration (including measures for regularities and color; cf. Table 1). We also used phase-scrambled images to study the role of lower-level visual processing on automatic evaluations of nature and urban scenes. Since color is relevant for naturalness perception and people have various associations with them (e.g., Palmer and Schloss, 2010; Kotabe et al., 2016; Schloss et al., 2017), we used color phase-scrambled images (in contrast to Valtchanov and Ellard, 2015). In such stimuli, the amplitude spectrum and therefore statistical regularity (i.e., scale invariance measured by the spectral slope) remained intact, while the depicted scene is no longer recognizable. Worthy of note is that edge information is, however, altered and therefore other relevant measures (e.g., self-similarity, fractal dimension) are influenced by the manipulation. Nevertheless, we decided to use these kinds of stimuli for several reasons. First, they are often used in visual perception research to control for lower-level processed properties. Second, they are appropriate to study the influence of the statistical regularities in the image (measured by the spectral slope) while controlling for recognizability. Third, they contain a larger range of diagnostic lower-level image properties (e.g., color) than more simplified control stimuli (as, for example, random noise patterns with a certain spectral slope). Finally, phase-scrambling has more advantages than other scrambling or control methods that destroy not only the spatial structure but also other relevant image characteristics. Similarly, it is preferable to other techniques (e.g., silhouette outlines) that are used to study fractal dimension (Table 1; e.g., Hagerhall et al., 2004; Patuano, 2018), because silhouette outlines allow at least some recognition of the scene or depicted objects. Therefore, phase-scrambled images are preferable to test whether lower-level processed image properties (including statistical regularities) influence the evaluation of nature and urban scenes when recognition is impeded.

The current work has therefore three major aims. First and foremost, we aimed to test contributions of characteristic lower-level processed image properties on affective and restoration-related associations and thereby to test the PFA. We sought to reveal implicit associations for image properties that are typical for nature and urban images by using phase-scrambled stimuli. Based on the literature reviewed above, we hypothesized that lower-level processing plays a role for the implicit associations with nature and urban images. This would be evident when the presentation of phase-scrambled images evoked associations similar to those of original images. Thereby, we also tested whether the finding of Valtchanov and Ellard (2015)—that phase-scrambled nature compared to urban images were preferred—could be replicated with color images in an implicit paradigm.

Second, we extended previous studies by including mood and stress restoration associations in addition to valence associations tested previously (cf., Joye et al., 2013; Sánchez et al., 2016; Mullin et al., 2017). Furthermore, we related the association strengths to a person’s connectedness to nature (CTN; Mayer and Frantz, 2004), because previous work showed that affective responses to natural and human-made stimuli are influenced by CTN (McMahan et al., 2018). In contrast to previous studies on implicit associations with nature, which contrasted effects for two environments, we used single-category IATs (SC-IATs; Karpinski and Steinman, 2006) to disentangle the associations for nature and urban images. In such SC-IATs, participants respond as fast as possible to presented stimuli, which were words of two evaluative attributes (e.g., “good” and “bad”) and images (e.g., phase-scrambled nature images). Associations between the images and the evaluative attributes are inferred from comparing reaction times for responding to the images in two different settings with the respective words (see Karpinski and Steinman, 2006, and Materials and Methods for further information). By using a SC-IAT instead of a traditional IAT, participants categorized the images not into two categories (i.e., “nature” and “urban”) but only as “image,” and therefore, the environmental categories were neither primed nor contrasted. Another reason for the use of an SC-IAT is the lack of spatial information in phase-scrambled images that makes it difficult—if not impossible—for the participants to categorize the images as “nature” or “urban,” which would be necessary to analyze IAT data.

Third, we extended previous studies on image properties by analyzing our stimuli for a comprehensive set of image properties that are known to differ between nature and urban scenes, correlate with naturalness, and/or are associated with affective evaluations (cf., e.g., Braun et al., 2013; Kardan et al., 2015; Table 1). Thereby, we compiled measures that were used in different areas of research, namely, landscape preference, empirical aesthetics, and vision science.

To sum up, in the current work, we investigated image properties in a set of nature and urban images. In experimental settings using a SC-IAT paradigm, we used not only original but also phase-scrambled stimuli, by which we were able to study lower-level processing independent of higher-level processing. Thus, we could measure effects of the preserved image properties on implicit associations with valence, mood, and stress restoration independent of content. We tested whether these preserved image properties (including the spectral slope) contribute to the evaluation of nature and urban images. We assume the spectral slope as an appropriate measure for internal regularity that leads to fluent and/or efficient processing, and therefore, we believe that the current work provides data relevant for current theories in the field, especially the PFA. The PFA would be supported if phase-scrambled nature and urban images lead to different implicitly measured evaluations.

## Study 1

This first study was designed as a pilot study for a larger study series to compile, analyze, and evaluate a stimulus set to investigate nature and urban scene perception. Therefore, this study includes and reports more stimulus types than were used in Study 2. As a first step, we created an image set of German natural and urban environments (as specified above). From the originals, a phase-scrambled version and line drawings (used for a different set of studies not reported here) were created. These images were analyzed for a comprehensive set of image properties and rated by participants. The relevance and description for the image properties are provided in Table 1. The aims of Study 1 were i) to select images for Study 2 and other study series not reported here, ii) to confirm that in the current picture set nature images were explicitly rated as more restorative than urban images, iii) to show that participants were unable to detect objects in the phase-scrambled images, and iv) to investigate image properties in the entire picture set and that used in Study 2. Therefore, an image set of 100 images per category (nature, urban) was rated for restorativeness. Phase-scrambled and line drawing versions of these 200 images were rated for recognizability of objects. Finally, image properties were analyzed and compared between nature and urban stimuli. Data on line drawings are presented in the Supplementary Material (Supplementary Tables 3, 6).

### Materials and Methods

#### Participants

In total, 104 participants rated the images. One participant was removed from the analyses because her responses for the object recognition task indicated that she was responding randomly. Of the remaining 103 participants, 79 participants indicated being female, 21 male, and three indicated either nothing or “other.” The age ranged between 18 and 35 years with a mean of 21.7 (*SD* = 2.8) years. Due to technical reasons, one participant had missing data for original images. This participant was excluded from analyses regarding original images only. All participants reported normal or corrected-to-normal vision. Most participants were students of the social sciences. They received either partial course credit or a ticket for the university cafeteria.

Following standard protocols in rating studies, data were analyzed item- and not participant-based because we intended to infer on differences between evaluations of image sets and not different response patterns based on the presentation of different stimulus sets. Therefore, we ran no *a priori*-power analysis but ensured to have a decent sample size. The item numbers (each 100; see below) are sufficient to detect a medium to small effect (*d* = 0.4) with a power of 0.8 and an alpha of 0.05 by using a two-tailed independent t-test.

#### Stimuli

We searched images on and downloaded them from colourbox.de, a stock photography page. Search terms were (in German): “Germany landscape,” “Germany nature,” “Germany city,” “Germany street,” and “Germany residence.” We avoided that an image contained humans, but if so they were in the background, rather small, and not prominent. Nature images did not depict humans or dominant human artifacts, such as streets or houses. If urban images contained green space, such as trees or grass, it was not dominant. We selected similar viewpoints for and diverse scenes of both nature and urban environments. The image list can be found in Supplementary Table 1 of the Supplementary Material. Images were cut to a square format and resized by bicubic interpolation to 512 × 512 pixels.

We created phase-scrambled images using Octave. By the use of Fast Fourier Transformation, a random phase was created and combined with the amplitude spectrum of each channel of the RGB color spectrum (e.g., Caddigan et al., 2017; Gong et al., 2018). Example images can be found in Figure 1. For information on the creation of line drawings and respective examples, see Supplementary Figure 2 and Supplementary Table 3 in the Supplementary Material.

#### Procedure

After participants signed informed consent, we introduced them to the experiment. First, they indicated whether and how well they could identify objects in the phase-scrambled images and line drawings using a 5-point scale ranging from “not at all” to “entirely.” All 400 stimuli (100 phase-scrambled nature images, 100 phase-scrambled urban images, 100 nature line drawings, 100 urban line drawings) were presented in random order to avoid frustration when completing this task on phase-scrambled images. Second, participants rated how restorative the original images were to them (“How restorative is this image for you?”; originally in German: “Wie erholsam wirkt das Bild auf Sie?”) using the same 5-point scale as for the previous rating. The 200 images (100 nature and 100 urban) were presented in random order. Third, we asked for demographic data. In total, the study lasted about 35 min. At regular intervals (during the ratings, every 100 trials), participants were allowed to take self-paced breaks to avoid fatigue.

A trial started with a black fixation cross on a gray screen (pixel value of 128). The duration of the fixation cross was random between 300 and 800 ms. Images were presented for one second and were then replaced by a gray screen with the rating scale.

Participants were tested in a lab with four computers, closed window blinds, and artificial light. Although participants were tested without chin rest, we asked them to keep the viewing distance constant at about 60 to 70 cm, which yields to a visual angle of approximately 11° for the height/width of the images. We calibrated the monitors (Dell P2214H, full HD 1,920 × 1,080 resolution) a few days before data collection using a Datacolor Spyder 5 Elite with the respective software (version 5.1). The settings were gamma = 2.2, temperature = 6,500 K, and luminance = 120 cd/m^2^. The procedure was conducted in line with the regulations of the German Psychological Society (DGPs) and the Declaration of Helsinki.

#### Image Analyses

We calculated image properties that are known to be associated with preference, naturalness, affective ratings, discomfort, and/or efficient coding (Table 1; see Supplementary Table 4 in the Supplementary Material for details on the calculation). They can be separated in measures that are related to color and brightness properties (mean and standard deviation [SD] of hue, saturation, and brightness), amplitude spectrum characteristics (spectral slope, weighted residuals, relative power in low, medium, and high spatial frequency ranges), and edge information (edge density, straight and non-straight edge density, anisotropy, complexity, self-similarity, fractal dimension, and entropy).

### Results

#### Rating Data

Original nature images (*M* = 4.31, *SD* = 0.87) were rated as more restorative than original urban images (*M* = 1.85, *SD* = 0.99; *t*(198) = 40.49, *p* < 0.001, *d* = 5.73). It was equally difficult to recognize objects in phase-scrambled nature (*M* = 1.20, *SD* = 0.50) and urban images (*M* = 1.20, *SD* = 0.50; *t*(198) = 0.24, *p* = 0.809, *d* = 0.03).

Stimuli for Study 2 were selected based on both high (nature) and low (urban) restorativeness ratings. We selected 20 original images per category (Supplementary Table 1 and Supplementary Figure 5). The original images used in Study 2 differed in their restorativeness ratings (*M*_*nature*_ = 4.21, *SD*_*nature*_ = 0.90, *M*_*urban*_ = 1.64, *SD*_*urban*_ = 0.85; *t*(38) = 24.37, *p* < 0.001, *d* = 7.08). Their phase-scrambled versions did not differ in their ratings on recognizability of objects (*M*_*nature*_ = 1.22, *SD*_*nature*_ = 0.51, *M*_*urban*_ = 1.21, *SD*_*urban*_ = 0.50; *t*(38) = 0.36, *p* = 0.724, *d* = 0.11).

#### Image Analyses

Detailed results of the image analyses for both all 100 images per category and the subset of 20 images per category can be found in Supplementary Table 6 and Supplementary Table 7 in the Supplementary Material. For original images (*n* = 100 each), spectral slope, weighted residuals, MSF, LSF, anisotropy, mean and SD of hue, mean and SD of saturation, fractal dimension, and overall, straight, and non-straight edge density differed between nature and urban images (|*t*|’s ≥ 4.08, *p*’s ≤ 0.001, |*d*|’s ≥ 0.58; Bonferroni-corrected *p*-threshold). Similarly, for the subset used in Study 2 (*n* = 20 each), spectral slope, weighted residuals, anisotropy, mean of hue, mean and SD of saturation, and overall, straight, and non-straight edge density differed between nature and urban images (|*t*|’s ≥ 3.93, *p*’s ≤ 0.001, |*d*|’s ≥ 1.24; Bonferroni-corrected *p*-threshold).

For phase-scrambled images (*n* = 100 each), spectral slope, weighted residuals, MSF, LSF, mean and SD of hue, mean and SD of saturation, fractal dimension, and overall, straight, and non-straight edge density were different for nature and urban images (|*t*|’s ≥ 3.78, *p*’s ≤ 0.001, |*d*|’s ≥ 0.53; Bonferroni-corrected *p*-threshold). Similarly, for the subset used in Study 2 (*n* = 20 each), spectral slope, weighted residuals, mean and SD of hue, mean and SD of saturation, and non-straight edge density were different for nature and urban images (|*t*|’s ≥ 3.69, *p*’s ≤ 0.001, |*d*|’s ≥ 1.17; Bonferroni-corrected *p*-threshold).

Due to having two image categories (leading to non-normal distribution of data and heteroscedasticity) and high collinearity of factors, we decided against regression analyses to calculate the predictive values of the image properties for restorativeness ratings of the 100 original images per category. However, to gain some information about the image properties’ relation to perceived restorativeness for original images, we run Spearman correlations: spectral slope, LSF, hue, mean and SD of saturation, and overall and non-straight edge density correlated positively with restorativeness (*r*_*s*_ ≥ 0.34, *p* < 0.001; Bonferroni-corrected *p*-threshold; Supplementary Table 8 in the Supplementary Material). Weighted residuals, HSF, MSF, anisotropy, SD of hue, and straight edge density correlated negatively with restorativeness ratings (*r*_*s*_ ≤ −0.23, *p* ≤ 0.001; Bonferroni-corrected *p*-threshold; Supplementary Table 8 in the Supplementary Material).

### Discussion

Study 1 aimed at replicating and extending previous findings on differing image properties for nature and urban images, higher restorativeness ratings for nature compared to urban images, and selecting images for the following experiment and another line of studies. We confirmed that nature images were rated as more restorative to view than urban images. Additionally, as expected due to the lack of spatial information, participants indicated very low—and similar for nature and urban images—ratings of recognizability for phase-scrambled images. Worthy of note is that each participant completed a total of 600 ratings, which may have affected the reliability of the ratings. However, such high trial numbers are common in the field (e.g., Berman et al., 2014; Spehar et al., 2015).

Similar to previous studies, several image properties differed between original nature and urban images. The spectral slope that represents statistical regularity and is associated with efficient coding, aesthetic perception, and visual discomfort (Table 1) was steeper for urban compared to nature scenes, as was reported previously (Braun et al., 2013). Relatedly, the weighted residuals were larger for urban than nature scenes, which is in line with the positive relationship of weighted residuals with rated discomfort in a previous study (Penacchio and Wilkins, 2015). These two findings confirmed “natural” amplitude spectrum characteristics in the current set of nature images, which were also reported previously for nature scenes (Burton and Moorhead, 1987; Ruderman and Bialek, 1994). Nature and urban images differed in their energy in the LSF and MSF, which may account for the differences in pleasure and cognitive load for filtered nature and urban images in previous work (Valtchanov and Ellard, 2015). As expected, urban scenes showed more straight edge and less non-straight edge density, which also contributed to higher anisotropy in such scenes compared to nature (Braun et al., 2013; Kardan et al., 2015). In contrast to Braun et al. (2013) and Wang and Ogawa (2015), the fractal dimension was higher for nature compared to urban scenes. Although fractal dimension and complexity are related, the difference for the latter was not significant, which was also true in Braun et al.’s study (2013). Differences in color were in line to a previous study that related them to naturalness (Kardan et al., 2015).

As expected, several image properties also differed between phase-scrambled nature and urban images. Especially, the amplitude spectrum and color measures differed in the same direction as for the original images. The results for the edge-related measures (e.g., anisotropy, edge densities) were different to those of original images, which is unsurprising because randomizing the phase information leads to distorted edge information. Overall, image analyses confirmed previous work and, therefore, strengthen the notion of differing lower-level processed properties in nature and urban scenes (and even in phase-scrambled versions). The rating and image analyses results of Study 1 justified the use of our stimulus set for the following study.

## Study 2

To test whether differences in image properties between nature and urban scenes evoke different automatic evaluations, we assessed implicit associations for original and phase-scrambled nature and urban images using SC-IATs. In a between-subjects design, we varied three attribute pairs: good and bad (valence), positive and negative mood, and restoration and stress. The attribute dimensions mood and stress restoration were chosen because nature and urban image presentation usually evokes changes in such outcomes. Valence was included to replicate and extend previous studies using standard IATs (Sánchez et al., 2016; Mullin et al., 2017). Overall, we assumed that original nature images would be associated with positive attributes while urban images would be associated with negative attributes. If the image properties present in phase-scrambled images contributed to such associations, a pattern similar to original images was expected.

### Materials and Methods

#### Participants

Initially, 287 participants—randomly assigned to the conditions—took part. Of these, 49 were excluded because they either aborted, were familiar with the study’s aim, had low overall accuracy (<0.75), indicated having red–green blindness, had no normal or corrected-to-normal vision, or stated afterward that they did not understand the instructions. The high dropout is mainly caused by two factors. First, we missed to inform participants beforehand to bring their glasses to ensure corrected-to-normal vision. Second, the study was part of a student’s project work that included to present the study aim and design to other students before data collection. However, despite telling these students that they should not attend, some of them did anyway. As they were not blind to the study’s hypotheses, we excluded them. Thus, the final sample consisted of 238 participants, who were evenly distributed across the conditions (78, 80, and 80 for valence, mood, and stress restoration, respectively). Their age ranged from 18 to 44 years (*M* = 21.8 years). Sixty-five participants indicated being male, 172 female, and one “other.” Most were students of the social sciences. Participants were relatively strongly connected to nature (measured by the connected to nature questionnaire; Mayer and Frantz, 2004; Cervinka et al., 2012), which was indicated by a slightly right-shifted histogram (Supplementary Figure 9 in the Supplementary Material) and a mean score of 45.48 on a 13- to 65-ranging scale. Participants received partial course credit.

Based on a previous study using nature and urban images in an IAT (Sánchez et al., 2016), we assumed an effect size of *f* = 0.31 and calculated the sample size *a priori* for a two-sided dependent t-test with α = 0.05 and power = 0.80. This analysis suggested 84 participants for each condition. Due to high dropout, the final sample size was slightly below the suggested one.

#### Stimuli

*Images*. We used 20 nature and urban images each in their original and phase-scrambled version (Study 1; Supplementary Table 1 and Supplementary Figure 5 in the Supplementary Material).

*Attribute words*. We compiled an initial word list during a practical course with students. It was based on word lists or questionnaires of related work (valence: Sánchez et al., 2016; mood: Watson et al., 1988; restoration: Korpela et al., 2008) and added with free associations by the students. This list consisted of 20 words for each attribute (good, bad, positive mood, negative mood, restoration, and stress). In a pilot study, words were rated online by 83 different participants on how well each word fit to its corresponding attribute (e.g., the word “peace” to the attribute “good”). For each attribute, we selected the 10 words with the highest ratings for the main study. The final word list can be found in Supplementary Table 9 in the Supplementary Material.

#### Design

Attribute dimension (valence, mood, stress restoration) was varied between subjects. Each participant completed four SC-IATs, one for each stimulus category (nature, urban) and image type (original, phase-scrambled). All participants started with the two SC-IATs containing phase-scrambled images to avoid associations with the assumed content of the images. Note that we advertised this study solely as a picture perception task and gave no information on the type of pictures. Thus, we could rule out that participants were systematically biased or expecting different environments when completing the task with the phase-scrambled images. Half of the participants started with nature, the other half with urban phase-scrambled images. Furthermore, we randomized the side of the response key for the images as well as the sides of the attributes between participants.

#### Procedure

**FIGURE 2:**
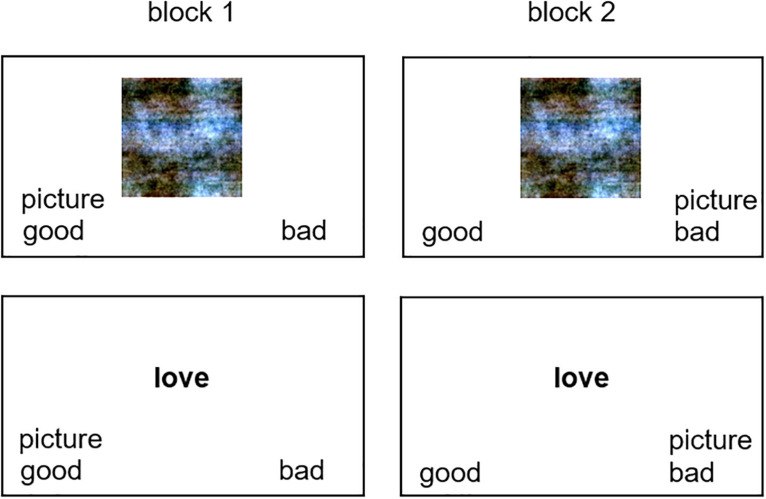
Example trials from the two blocks in a given SC-IAT (here: phase-scrambled images with valence attributes). Note that for better visibility, the size of text indicating the response keys is depicted larger than it was during the experiment.

Setup and procedure of each SC-IAT were based on Karpinski and Steinman (2006). In contrast to their procedure, we used 20 images and 10 attribute words each as stimuli. Therefore, presentation frequency (to ensure that both response keys were correct equally often) was different, namely, 1:1:3. In short, the procedure of the SC-IATs was as follows. After giving their informed consent, participants were introduced to the specific task (i.e., responding to words and images with the indicated keys as accurate and fast as possible), response keys (“A” and “L” on a QWERTZ keyboard), and stimuli including their correct assignments to the keys in a given block (e.g., any image to “picture”; the word “peace” to “good”; the word “poverty” to “bad”). Stimuli were presented individually and centrally until a response was recorded (Figure 2). We asked participants to press the predefined key as fast as possible. Note that this was not an evaluation task but that there was always a correct response: Participants were asked to respond to images always with the key for “picture,” to good words with the key for “good” and so on (Figure 2). They received feedback on their accuracy (green “o” for correct, red “x” for incorrect, and [in German] “please respond faster” when they responded after 1,500 ms after stimulus onset). The attribute words and images were presented at random order within a given block.

Within each SC-IAT, participants completed two blocks with 96 trials each (including 24 training trials which were excluded for the analyses), in which we asked them to respond to a presented image with the same key as to positive words or to negative words, respectively (Figure 2). Based on reaction times in the two blocks, association strengths between the images and the two attributes (e.g., “good” and “bad”) were measured.

After completing the SC-IATs, we collected data on the participants’ CTN, which was surveyed using the original CTN questionnaire (Mayer and Frantz, 2004) in a German version that was previously used by Cervinka et al. (2012). The reliability of the scale was good (Cronbach’s α = 0.82). The study was conducted in accordance with the German Psychological Society (DGPs) and the Declaration of Helsinki. It was approved by the local ethics committee of the university where the study was planned and conducted.

Data collection was in a different lab than in Study 1 but under comparable conditions. Monitors (LG 22MB35, full HD 1,920 × 1,080 resolution) were calibrated with the same settings as in Study 1. Viewing distance was about 100–110 cm, which resulted in a visual angle of approximately 7° for the height and width of the images.

#### Data Preprocessing and Analyses

We preprocessed data as recommended by Karpinski and Steinman (2006). In brief, we considered only trials with reaction times between 350 and 1,500 ms. We substituted values of incorrect trials by the average reaction time in the given block plus 400 ms. Mean reaction times of the block in which images were paired with positive attributes (good, positive mood, restoration) were subtracted from those blocks in which images were paired with the negative attributes (bad, negative mood, stress), and this value was then divided by the standard deviation of all correct responses in both blocks. We used these D-scores in the statistical analyses. A positive (negative) D-score represents faster responses in blocks where participants needed to respond with the same key for images and good (bad), positive (negative) mood, and restoration (stress) and therefore indicates the respective association of the image category (e.g., nature original images) with these attributes (e.g., “good”). D-scores can range between −2 and +2. To test whether D-scores were different from zero and thus to determine whether there is a significant bias (i.e., association) for a given image set to one of the two attributes, we used two-sided one-sample t-tests. Additionally, we tested with two-sided dependent t-tests whether the D-scores differed for nature and urban images.

### Results

#### Original Images

**FIGURE 3:**
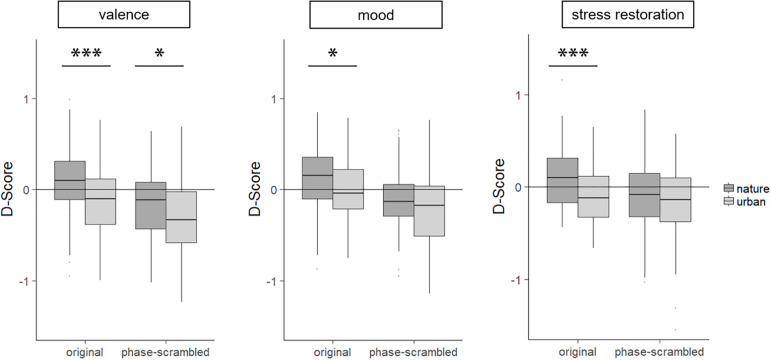
Results of Study 2 represented as box plots. A positive D-score indicates an association of the image category with good, positive mood, and restoration. A negative D-score indicates an association of the image category with bad, negative mood, and stress. D-scores can vary between −2 and +2. Asterisks indicate significance of t-tests comparing nature and urban images. For one-sample t-tests testing against zero, see main Table 2. ^∗^*p* < 0.05, ^∗∗∗^*p* < 0.001.

One-sample t-tests on the D-scores indicated that original nature images were associated with positive attributes (“good,” “positive mood,” and “restoration”), while urban images were associated with “bad” and “stress” (Figure 3 and Table 2). D-scores for original urban images were not different from zero for the mood condition, indicating to clear association to either “positive mood” or “negative mood.” In all attribute conditions, D-scores differed between original nature and urban images, indicating that different associations were evoked by these environments (Figure 3 and Table 2). For participants in the mood condition, we found a positive correlation between CTN and the D-score for original nature images (*r*(78) = 0.29, *p* = 0.010, significant also for Bonferroni correction of *p*-threshold *p* = 0.0125). All other correlations were non-significant (*p*’s ≥ 0.090).

**TABLE 2:** Mean (*M*) and standard deviation (*SD*), as well as results from one-sample *t*-tests against zero and dependent *t*-tests for the comparison of nature and urban images for original and phase-scrambled images in Study 2.

					**One-sample t-test**			**Dependent t-test**		
			***M***	***SD***	***t***	***p***	***d***	***t***	***p***	***d***
**Valence (*n* = 78)**										
	**Original**									
		Nature	0.09	0.37	2.06	0.043	0.23	3.62	<0.001	0.41
		Urban	−0.12	0.35	−2.95	0.004	0.33			
	**Phase-scrambled**									
		Nature	−0.17	0.38	4.02	<0.001	0.46	2.19	0.032	0.25
		Urban	−0.30	0.38	6.90	<0.001	0.78			
**Mood (*n* = 80)**										
	**Original**									
		Nature	0.12	0.34	3.02	0.003	0.34	2.23	0.028	0.25
		Urban	−0.01	0.36	0.12	0.909	0.01			
	**Phase-scrambled**									
		Nature	−0.13	0.37	3.05	0.003	0.34	1.69	0.096	0.19
		Urban	−0.21	0.39	4.90	<0.001	0.55			
**Stress restoration (*n* = 80)**										
	**Original**									
		Nature	0.10	0.32	2.63	0.010	0.29	3.97	<0.001	0.44
		Urban	−0.10	0.32	−2.85	0.006	0.32			
	**Phase-scrambled**									
		Nature	−0.09	0.35	2.35	0.021	0.26	1.26	0.210	0.14
		Urban	−0.16	0.39	3.69	<0.001	0.41			

*A positive mean value indicates an association of the image category with good, positive mood, or restoration; a negative mean value indicates an association of the image category with bad, negative mood, or stress.*

#### Phase-Scrambled Images

All phase-scrambled images, irrespective of depicted environment, were associated with negative attributes (i.e., “bad,” “negative mood,” and “stress”; Figure 3 and Table 2). D-scores and thus association strengths for phase-scrambled nature and urban images were different for valence but not for mood or stress restoration (Table 2). CTN did not correlate with any of the D-scores for the phase-scrambled images (*p*’s ≥ 0.289).

### Discussion

To our knowledge, this study is the first to show implicitly measured associations of nature with positive mood and restoration and of urban environments with stress. The results were in line with experimental findings on positive effects evoked by viewing nature compared to urban environments (e.g., Hartig et al., 1996; Berman et al., 2008; Gladwell et al., 2012; Valtchanov and Ellard, 2015). Replicating previous findings, original nature images were associated with positive valence, while urban images were associated with negative valence, even when they were not contrasted with the other environment (cf., van der Ha, 2011; Sánchez et al., 2016; Mullin et al., 2017; Beute and de Kort, 2019). In general, association strengths were rather small.

The non-significant association of urban images with negative mood indicates that urban scenes were evaluated either neutral or ambivalent. When considering the effect sizes for the comparison of nature and urban images, the mood condition led to smaller effects than the other two conditions. Relatedly, previous lab studies failed to find effects on mood after viewing images (Berman et al., 2008; Gamble et al., 2014). Although a meta-analysis confirmed emotion effects after the exposure to nature, the authors found slightly smaller effect sizes for lab compared to *in situ* studies (McMahan and Estes, 2015). Moreover, mood can be considered the most specific attribute dimension within the current study (as it can be considered as a part of restoration) and therefore it was likely to find smaller effects for mood than for the other dimensions. Note that in the current study, the mood words were adjectives rather than nouns as in the other two conditions. This might have had an influence on the results, although we are not aware of similar patterns in the literature.

The association strength of original nature images with mood-related attributes correlated moderately with the CTN. Similarly, McMahan et al. (2018) found that CTN moderates the affective change after nature exposure. Thus, it seems that CTN specifically is associated with affective benefits of nature exposure while valence- and restoration-related evaluations are not.

The current study is also the first to investigate implicitly measured associations evoked by phase-scrambled nature and urban images. For phase-scrambled images, irrespective of category and attribute dimension, we found D-scores significantly below zero, indicating associations with the negative attributes (bad, negative mood, stress). Thus, spatial information and higher-level cognitive processing (i.e., recognition of objects) seem necessary for positive associations. Similarly, with the use of different scrambling techniques, Kotabe et al. (2017) showed that recognition of the depicted scene is necessary to find a preference for nature stimuli. This is also in line with previous work on natural and urban sounds which showed that the preference for nature sounds was eliminated when recognition of the source (i.e., nature or urban) was impeded (Van Hedger et al., 2019).

For the attribute dimensions of mood and stress restoration, no differences between phase-scrambled nature and urban images were found. However, urban phase-scrambled images were associated more strongly with negative valence than nature phase-scrambled images. Thus, valence association strengths differed between nature and urban phase-scrambled images, although both categories were associated with “bad.” This corresponds to the finding of higher preference for phase-scrambled nature compared to urban images (Valtchanov and Ellard, 2015). Therefore, we conclude that differences in properties (e.g., color, spectral slope) between phase-scrambled nature and urban images led to different association strengths for valence. We suppose that such differences in image properties contribute to the associations evoked by original images. The finding that phase-scrambled images of two categories affected different associations is also relevant for studies that use such stimuli as a control or visual mask. Authors of such studies need to keep in mind that the associations evoked by phase-scrambled images might be category-specific and thus bias responses.

## General Discussion

In two studies, we investigated the role of lower-level processing of nature and urban stimuli with regard to their effects on restoration-related associations. Study 1 confirmed that several lower-level processed image properties differ between nature and urban scenes (e.g., Braun et al., 2013; Kardan et al., 2015; Penacchio and Wilkins, 2015). Study 2 revealed that (original) nature images are associated with positive mood and restoration and replicated that nature images are associated with positive valence (Beute and de Kort, 2019; Mullin et al., 2017; Sánchez et al., 2016). Thus, the positive associations for original nature images are in line with the theories in the field (ART, SRT, and PFA), which assume higher preference, positive affective states, and/or restoration for nature sceneries (Ulrich, 1983; Kaplan, 1995; Joye and van den Berg, 2011).

Phase-scrambled images—which lack the spatial information while preserving several lower-level processed properties—were associated with negative valence, negative mood, and stress. Only for valence did we record a difference between nature and urban phase-scrambled images, indicating that the lower-level image properties contribute to the association found for the original images. Since these stimuli lack spatial information, recognition is impeded (see also Study 1), and therefore the difference must have been due to the different image properties, which in turn led to different associations with valence. These diverging associations could have several sources, e.g., fluent processing of nature images leading to a more positive affective state and therefore an association with positive valence (as proposed by the PFA; Joye and van den Berg, 2011) or by different colors evoking different association networks and therefore different valence associations (cf., Palmer and Schloss, 2010). Further studies with appropriate and well-controlled stimuli (e.g., random patterns with specific spectral slopes or uniform color patches) are necessary to disentangle the roles of the different image properties for the found effects.

The finding that phase-scrambled nature and urban images were associated differently with the attribute dimension of valence but not restoration points in the direction of the idea discussed by Valtchanov and Ellard (2015) that affective evaluation and processing load (measured by blink rates in their study) might be two separate mechanisms. However, the null effect for mood contradicts this conclusion, albeit it is noteworthy that the mood effect was the smallest for the original images. Specifically designed studies are needed to address the question of separate mechanisms and the roles of lower-level visual processing for these two mechanisms (cf. Valtchanov and Ellard, 2015). Worthy of note is that the use of an implicit measure allowed us to study automatic evaluations without the need of participants’ explicit attribution of the evaluation terms to the stimuli. Therefore, the negative findings for the attribute dimensions of mood and restoration for the phase-scrambled images cannot be caused by the participants’ inability to attribute the stimuli to these evaluative dimensions.

The findings of differing image properties between nature and urban images, the correlations of restorativeness and image properties related to efficient coding, and the difference in valence association strength for nature and urban phase-scrambled images indicated at least some support for the PFA. However, since the difference in valence association was rather small and not significant for the other attribute dimensions, the current results are still inconclusive and future studies are needed to test the PFA further and more directly.

### Limitations

Our implications on the PFA are based on the assumption that the spectral slope is a suitable measure for regularity that assumingly leads to fluent processing and effects claimed by the PFA. As indicated in the introduction and in Study 1, the spectral slope is not the only measure related to regularity or fractal characteristics. Phase-scrambling destroyed edge properties and therefore characteristics that are measured by fractal dimension and self-similarity. In a related line of research, the relation of edge properties to perceived naturalness, aesthetic perception, and higher-order cognitive function is also studied (e.g., Berman et al., 2014; Kardan et al., 2015; Schertz et al., 2018, 2020). This work shows that, for images in which one cannot recognize the content, edge characteristics can explain some effects found for original images (Schertz et al., 2020), but not preference (Kotabe et al., 2017). Therefore, more research and further manipulation techniques are needed to investigate affective and restorative effects of regularity and internal repetition independent of content while also considering potential effects of edge properties.

In the current work, we focused on automatic associations of nature and urban stimuli with three evaluation dimensions using SC-IATs. Although this approach is useful, its explanatory power is limited. Explicit evaluations and restoration measures after exposure to such stimuli are also needed. In a related study series where participants viewed such images in a classic restoration paradigm (cf., Berman et al., 2008; for reviews, see Ohly et al., 2016; and Stevenson et al., 2018), we investigated effects of viewing original and phase-scrambled images on mood, working memory, and other measures (Menzel and Reese, 2021). We also included stimuli that were controlled for lower-level properties while enabling higher-level processing. The results of this study series support the finding from the current study that spatial information and thus recognition of the environment is necessary for the beneficial outcomes associated with viewing nature compared to urban environments (Menzel and Reese, 2021; see also Kotabe et al., 2017; and Van Hedger et al., 2019, for related findings). Nevertheless, the current state of research does not allow concrete conclusions about the validity of the PFA.

Worthy of note is that we used a dichotomous stimulus set and therefore disregarded scenes not fitting in our categories of “nature” and “urban.” Especially, potentially restorative urban scenes including those frequently used for social encounters, such as cafés and restaurants, shopping malls, surroundings with both built and natural elements, museums, or private indoor environments, are excluded from the current set of stimuli. We agree with aspirations and calls to consider also restorative elements in urban surroundings, context and actual need for restoration, and social aspects in research on restorative environments (e.g., Staats et al., 2016; Weber and Trojan, 2018). Nevertheless, our study was designed to investigate the role of lower-level processing on the perception of such dichotomous stimulus sets, rather than identifying further sources of restoration or factors influencing them.

### Concluding Remarks

The current studies showed that image properties differ between nature and urban images and that they contributed to associations with valence, as corresponding D-scores were different for nature and urban phase-scrambled images. However, the results clearly showed that spatial information—and therefore the recognition of the environment—is necessary to evoke positive associations with nature, as all D-scores were negative for phase-scrambled images but not for original images. Based on current and previous findings, we assume that it is likely that lower-level processing plays a mediating or moderating role for restoration, while higher-level processing seems necessary to evoke similar (and positive) associations as with the original images.

## Data Availability Statement

The raw data supporting the conclusions of this article will be made available by the authors, without undue reservation.

## Ethics Statement

The studies involving human participants were reviewed and approved by the Local Ethics Committee of Department 8 of the University of Koblenz-Landau, Institute of Psychology, University of Koblenz-Landau, Fortstraße 7, Landau (Pfalz), Germany. The patients/participants provided their written informed consent to participate in this study.

## Author Contributions

CM designed and planned all studies, supported by GR. CM supervised data collection of all studies, analyzed all data, and wrote the manuscript. Both authors contributed to the final version of the manuscript.

## Conflict of Interest

The authors declare that the research was conducted in the absence of any commercial or financial relationships that could be construed as a potential conflict of interest.
