# Supplementary material for: Implicit Associations With Nature and Urban Environments: Effects of Lower-Level Processed Image Properties
Source: Front Psychol. 2021 May 20;12:591403. doi: 10.3389/fpsyg.2021.591403 (PMC8172784; doi:10.3389/fpsyg.2021.591403)
Supplement: Supplementary file 1 [file Data_Sheet_1.docx]

**Supplemental Material to Menzel & Reese: “Implicit associations with nature and urban environments: Effects of lower-level processed image properties”**

Table S1. Images used in Studies 1 (no indicator) and 2 (^+^). Images can be accessed on colourbox.de using the noted ID.

| category | image ID |
| --- | --- |
| nature | 10015121, 10015238, 10015361^+^, 10053273^+^, 10091542, 1025356, 10441262, 10485567, 10508084, 10710818, 10835093, 10894081^+^, 10894098^+^, 10949756, 11007330, 11231656, 11435318, 11512024, 11512041, 11618880^+^, 1207683, 12585176, 12935962, 13565744, 13742919, 13844724^+^, 17796440^+^, 18103350, 1948285, 20212943, 21578564, 2185835, 22824806, 2352894, 2365871^+^, 2480953, 2488947^+^, 2607912, 2608039, 2608135, 2729142, 2729438, 2760543, 2922966, 3023541, 3179208, 3378328^+^, 3466480^+^, 3595156, 3788920^+^, 3788921, 3802211, 3846072, 4005221^+^, 4021164, 4427411, 4569194, 4569206, 4583660, 4657696, 4716064^+^, 5100417, 5130886, 5359207, 5368617, 5368736, 5368843, 5369070, 5369508, 5369566, 5804285^+^, 6165182, 6165825^+^, 6255146, 6261444, 6381281, 6382750, 6422149, 6424738, 6425101^+^, 6425181^+^, 6591934^+^, 6669704, 6783516, 7165486, 7223741, 7531206, 7794939, 7935139^+^, 7966162, 8117048, 8155781, 8192114, 8294139, 9502254, 9600960, 9600968, 9603897, 9740938, 9759623 |
| urban | 10015182, 1007574, 1007777, 10361401, 10378832, 10384378, 10547957^+^, 10730291, 1076160, 1076222^+^, 11322731, 11597067^+^, 11601356, 11756008, 11850666, 11890378, 11975512, 11999198, 1208257, 1208259^+^, 12123851, 12190299, 12196268, 12693132, 1290900, 13089236, 13188561, 13212989, 13334757, 13640953, 13640987, 13641014, 13641023, 14272719^+^, 14272743, 14272772, 14276706, 14972994, 15324703, 15747130, 1586138, 15994176, 16306377, 16844021, 17346113, 17574162, 17710049, 17998745, 18131360^+^, 18556902, 18666791, 1922428, 20995949, 2235448, 22926446, 22993760, 22993898, 22993911, 22993954, 23485686^+^, 2612104^+^, 3194143, 5358369^+^, 5498772^+^, 6121632, 6367694, 6421804, 6421828^+^, 6421909, 6422118, 6430243^+^, 6430343^+^, 6430431^+^, 6446477^+^, 6535183, 6596422, 6596616, 6906876, 7134857^+^, 7153700, 7153742, 7172760^+^, 7231339, 7567159^+^, 7626629, 7682405, 7732320^+^, 7732384^+^, 7999826, 8094709, 8128585, 8148463, 8203306, 8380980, 854230, 9515130, 953999, 9599523, 9629387, 9743295 |

Figure S2. Examples of original, phase-scrambled, and line drawing stimuli of nature and urban environments used in Study 1. Original images were from colourbox.de and cropped to a square format.


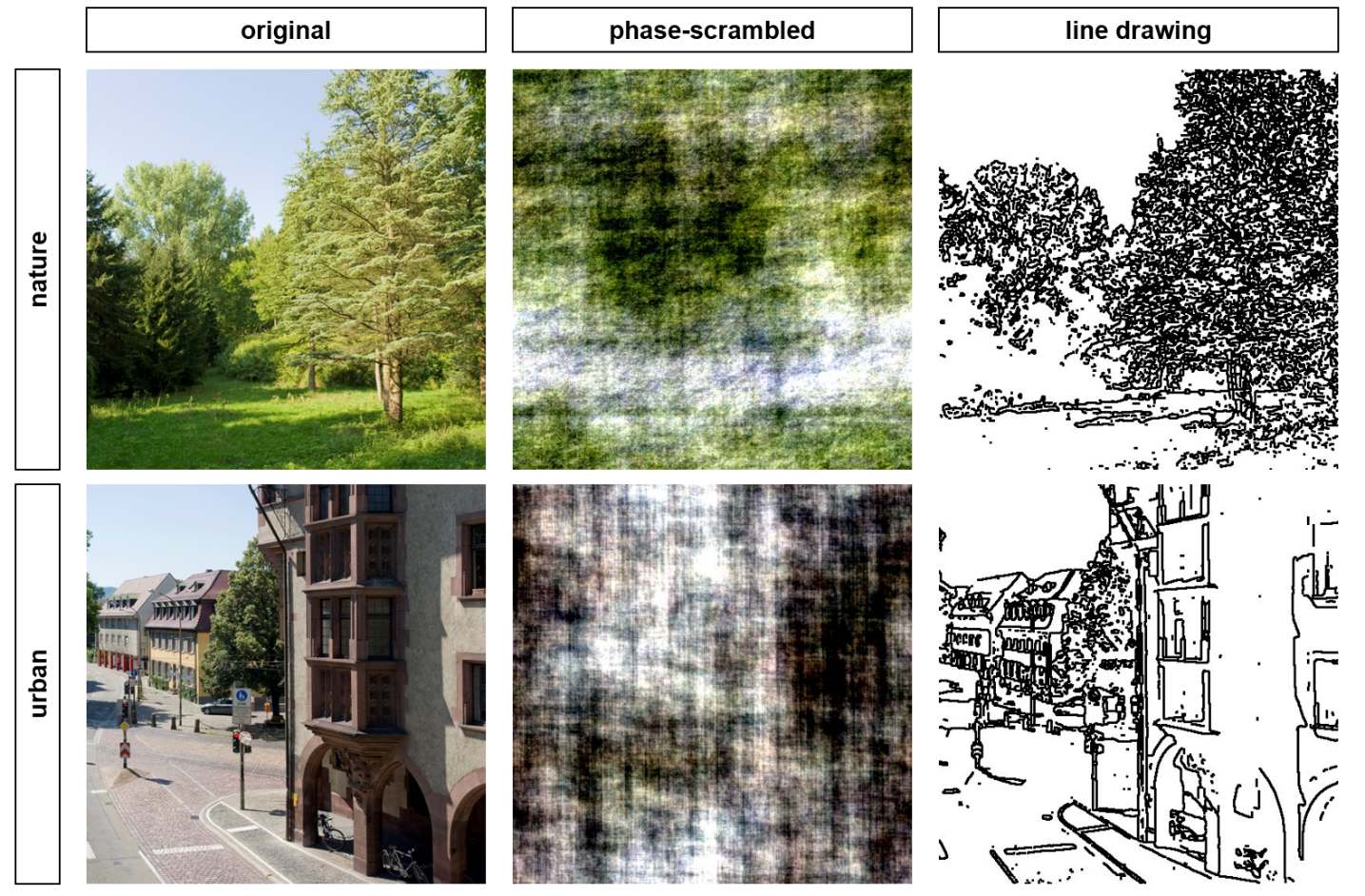


Table S3. Information on line drawings (Study 1).

|  | Explanation |
| --- | --- |
| Creation of stimuli | Line drawings were created using the following procedure in ImageJ. First, a Gaussian blur with a sigma of 1 was applied. Second, edges were identified using a Sobel edge detector. Third, a binary image was created. Fourth, in this binary image, pixels were removed from the edges until they are reduced to single-pixel-wide shapes (‘Skeletonize’ function). Finally, using the ‘Dilate’ function pixels were added to magnify the lines evenly. Example images can be found in Figure S2. |
| Results of recognition rating | It was easier to recognize objects in urban scenes (*M* = 4.37, *SD* = 0.75) than nature scenes (*M* = 3.54, *SD* = 1.03; *t*(198) = 20.76, *p* < .001, *d* = 2.94). |
| Results of image analyses | For the 100 line drawings per category, spectral slope, MSF, complexity, anisotropy, mean and SD of brightness, entropy, fractal dimension, as well as overall, straight, and non-straight edge density differed between nature and urban images (\|*t*\|’s ≥ 3.14, *p*’s ≤ .002, \|*d*\|’s ≥ 0.44). For more information, see Table S6. |

Table S4. Image properties calculated for Study 1.

| image property | calculation |
| --- | --- |
| spectral slope | slope of the curve of the radially-averaged spatial frequency and spectral power (i.e., amplitude squared) of an image in a log-log plot: input images were 512 × 512 pixels; custom-written script for python provided by Oliver Langner; images converted to Lab colour space; L channel is used for further analyses; fast Fourier transformation; spectral slope is the slope of the 1D power spectrum (log-log plot of power and spatial frequency) between 10 and 255 pixels (calculated using 33 bins) |
| weighted residuals | deviation of the amplitude spectrum compared to a modelled 2D amplitude spectrum with a slope of -1 in the 1D spectrum, weighted by spatial frequency sensitivity, and adjusted by the differing energy in horizontal and vertical orientations (Penacchio & Wilkins, 2015): input images were 256 × 256 pixels (to match the modelled amplitude spectrum); custom-written script for Matlab provided by Olivier Penacchio; contrast sensitivity function was adjusted to the image presentation size in Study 1 and 3 (i.e., 11°); for further information on the procedure see Penacchio & Wilkins (2015) |
| HSF | sum of the power of high (> 24 cpi) spatial frequencies divided by power of all frequencies: input images were 512 × 512 pixels; custom-written script for python provided by Oliver Langner; images converted to Lab colour space; L channel is used for further analyses; fast Fourier transformation; power (amplitude squared) of > 24 to 512 cycles per image divided by overall power of the image. |
| MSF | sum of the power of medium (8 - 24 cpi) spatial frequencies divided by of all frequencies: input images were 512 × 512 pixels; custom-written script for python provided by Oliver Langner; images converted to Lab colour space; L channel is used for further analyses; fast Fourier transformation; power (amplitude squared) between 8 to 24 cycles per image divided by overall power of the image. |
| LSF | sum of the power of low (< 8 cpi) spatial frequencies divided by of all frequencies: input images were 512 × 512 pixels; custom-written script for python provided by Oliver Langner; images converted to Lab colour space; L channel is used for further analyses; fast Fourier transformation; power (amplitude squared) of < 8 cycles per image divided by overall power of the image. |
| self-similarity | similarity of gradient histograms of sub-images and the entire image (e.g., Braun et al., 2013): input images were 512 × 512 pixels; custom-written script for Matlab provided by Christoph Redies; gradient histograms were calculated as described in Braun et al. (2013) for the entire image (level 0) and compared to the sub-images at level 3 (therefore, the source image is divided into four [level 1], then sixteen [level 2] and finally 64 [level 3] sub-images). |
| complexity | sum of gradient strengths (e.g., Braun et al., 2013): input images were 512 × 512 pixels; custom-written script for Matlab provided by Christoph Redies; calculation of gradient histogram as described in Braun et al. (2013) |
| anisotropy | distribution of gradient orientations (e.g., Braun et al., 2013): input images were 512 × 512 pixels; custom-written script for Matlab provided by Christoph Redies; calculation of gradient histograms in 16 bins for 360° as described in Braun et al. (2013) |
| hue | average dimension of colour (e.g., Berman et al., 2014; Kardan et al., 2015): input images were 512 × 512 pixels; custom-written script for Matlab provided by Omid Kardan; images converted to HSV colour channel; average hue across all pixels |
| saturation | average saturation (e.g., Berman et al., 2014; Kardan et al., 2015): input images were 512 × 512 pixels; custom-written script for Matlab provided by Omid Kardan; images converted to HSV colour channel; average saturation across all pixels |
| brightness | average value of the colour (e.g., Berman et al., 2014; Kardan et al., 2015): input images were 512 × 512 pixels; custom-written script for Matlab provided by Omid Kardan; images converted to HSV colour channel; average brightness across all pixels |
| SD of hue | diversity of colour; standard deviation of hue across all pixels (e.g., Berman et al., 2014; Kardan et al., 2015): input images were 512 × 512 pixels; custom-written script for Matlab provided by Omid Kardan; images converted to HSV colour channel; average standard deviation of hue across all pixels of an image |
| SD of saturation | standard deviation of saturation across all pixels (e.g., Berman et al., 2014; Kardan et al., 2015): input images were 512 × 512 pixels; custom-written script for Matlab provided by Omid Kardan; images converted to HSV colour channel; standard deviation of saturation across all pixels |
| SD of brightness | standard deviation of all pixel values; similar to the contrast of an image (e.g., Berman et al., 2014; Kardan et al., 2015): input images were 512 × 512 pixels; custom-written script for Matlab provided by Omid Kardan; images converted to HSV colour channel; standard deviation of brigthness across all pixels |
| entropy | uniformity of intensity histogram; similar to the randomness of an image (e.g., Berman et al., 2014; Kardan et al., 2015): input images were 512 × 512 pixels; custom-written script for Matlab provided by Omid Kardan; greyscale histogram with 256 bins; calculation of entropy using $Entropy=-\sum_{n=1}^{256} \left( p_{n}*{log}_{2}p_{n} \right)$ |
| edge density | number of pixels on edges divided by the total number of pixels (e.g., Berman et al., 2014; Kardan et al., 2015): input images were 512 × 512 pixels; custom-written script for Matlab provided by Omid Kardan; Canny edge detection based on various thresholds as described in Berman et al. (2014); number of pixels on edges divided by total number of pixels |
| straight edge density | number of pixels on straight edges divided by the total number of pixels (e.g., Berman et al., 2014; Kardan et al., 2015): input images were 512 × 512 pixels; custom-written script for Matlab provided by Omid Kardan; Canny edge detection based on various thresholds as described in Berman et al. (2014); gradient-based connected component algorithm to detect straight lines in the image as described in Berman et al. (2014); number of pixels on straight edges divided by total number of pixels |
| non-straight edge density | number of pixels on non-straight edges divided by the total number of pixels (e.g., Kardan et al., 2015): input images were 512 × 512 pixels; custom-written script for Matlab provided by Omid Kardan; Canny edge detection based on various thresholds as described in Berman et al. (2014); gradient-based connected component algorithm to detect straight lines in the image as described in Berman et al. (2014); number of pixels on non-straight edges divided by total number of pixels |
| fractal dimension | complexity measured in binary image versions by the Boxcount method (Braun et al., 2013): input images were 512 × 512 pixels; custom-written script for Matlab; images were converted to Lab colour space; L channel is used for further analyses; Canny Edge filter with threshold of 0.2 for binarization; image is then resized to the area in which structure is existing; for varying sizes, a box is checked (and counted) on whether pixel values are above 0; relation of box size and counted boxes is calculated in a log-log plot; linear regression from this relationship; slope of the curve is measured fractal dimension |

Figure S5. Original (left) and phase-scrambled (right) images of nature (top) and urban (bottom) environments used in Study 2. Original images were from colourbox.de and cropped to a square format.


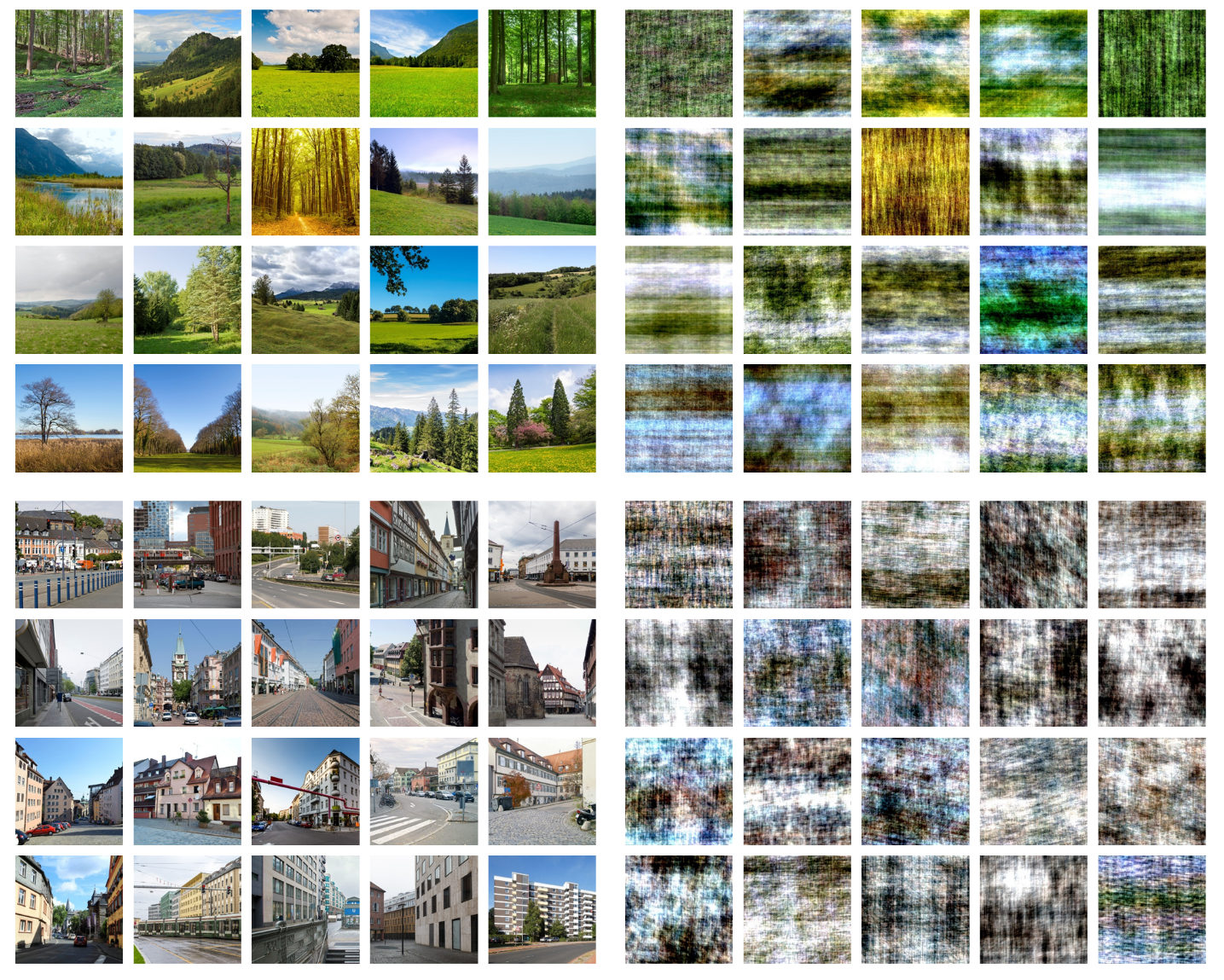


Table S6: Mean values of image measures for the entire image set (*n* = 100 per image category). Results of *t*-test (df=198) comparing nature and urban images. Rows in bold indicate significant difference between nature and urban images (critical *p*-value based on Bonferroni-correction: *p* = .0026).

| image type | image property | image category | | results of *t*-test | | |
| --- | --- | --- | --- | --- | --- | --- |
|  |  | nature | urban | *t* | *p* | *d* |
| original | **spectral slope** | **-2.23** | **-2.42** | **7.35** | **< .001** | **1.04** |
|  | **weighted residuals** | **3.54*10^11^** | **6.58*10^11^** | **-11.09** | **< .001** | **-1.57** |
|  | HSF | 0.008 | 0.013 | -2.96 | .003 | -0.42 |
|  | **MSF** | **0.025** | **0.041** | **-4.43** | **< .001** | **-0.63** |
|  | **LSF** | **0.967** | **0.946** | **4.08** | **< .001** | **0.58** |
|  | self-similarity | 0.60 | 0.56 | 2.37 | .019 | 0.34 |
|  | complexity | 12.33 | 11.51 | 1.50 | .134 | 0.21 |
|  | **anisotropy** | **4.09*10^-4^** | **6.14*10^-4^** | **-12.18** | **<.001** | **-1.72** |
|  | **hue** | **1.40** | **-0.16** | **6.97** | **< .001** | **0.99** |
|  | **saturation** | **0.46** | **0.28** | **9.24** | **< .001** | **1.31** |
|  | brightness | 0.59 | 0.63 | -2.75 | .006 | -0.39 |
|  | **SD of hue** | **1.23** | **1.54** | **-4.72** | **< .001** | **-0.67** |
|  | **SD of saturation** | **0.25** | **0.20** | **5.75** | **< .001** | **0.81** |
|  | SD of brightness | 0.25 | 0.25 | -1.14 | .257 | -0.16 |
|  | entropy | 7.52 | 7.47 | 1.23 | .221 | 0.17 |
|  | **edge density** | **0.13** | **0.10** | **10.94** | **< .001** | **1.55** |
|  | **straight edge density** | **0.07** | **0.13** | **-12.56** | **< .001** | **-1.78** |
|  | **non-straight edge density** | **268.90** | **61.58** | **22.25** | **< .001** | **3.15** |
|  | **fractal dimension** | **1.74** | **1.70** | **4.09** | **< .001** | **0.58** |
| phase-scrambled | **spectral slope** | **-2.26** | **-2.42** | **6.01** | **< .001** | **0.85** |
|  | **weighted residuals** | **3.08*10^11^** | **5.71*10^11^** | **-11.23** | **< .001** | **-1.59** |
|  | HSF | 0.008 | 0.011 | -2.57 | .011 | -0.36 |
|  | **MSF** | **0.024** | **0.037** | **-4.25** | **< .001** | **-0.60** |
|  | **LSF** | **0.968** | **0.952** | **3.82** | **< .001** | **0.54** |
|  | self-similarity | 0.92 | 0.92 | 1.58 | .116 | 0.22 |
|  | complexity | 15.22 | 16.38 | -2.13 | .034 | -0.30 |
|  | anisotropy | 1.61*10^-4^ | 1.68*10^-4^ | -0.77 | .442 | -0.11 |
|  | **hue** | **1.49** | **-0.27** | **7.38** | **< .001** | **1.04** |
|  | **saturation** | **0.39** | **0.24** | **8.60** | **< .001** | **1.22** |
|  | brightness | 0.57 | 0.60 | -2.85 | .005 | -0.40 |
|  | **SD of hue** | **0.99** | **1.37** | **-5.81** | **< .001** | **-0.82** |
|  | **SD of saturation** | **0.25** | **0.20** | **8.31** | **< .001** | **1.18** |
|  | SD of brightness | 0.23 | 0.24 | -0.23 | .816 | -0.03 |
|  | entropy | 7.74 | 7.74 | 0.08 | .940 | 0.01 |
|  | **edge density** | **0.17** | **0.17** | **4.88** | **< .001** | **0.69** |
|  | **straight edge density** | **0.04** | **0.06** | **-5.32** | **< .001** | **-0.75** |
|  | **non-straight edge density** | **482.91** | **401.44** | **9.09** | **< .001** | **1.29** |
|  | **fractal dimension** | **1.96** | **1.95** | **3.78** | **< .001** | **0.53** |
| line drawing | **spectral slope** | **-1.77** | **-1.95** | **12.28** | **< .001** | **1.74** |
|  | weighted residuals | 4.04*10^12^ | 4.36*10^12^ | -2.09 | .037 | -0.30 |
|  | HSF | 0.062 | 0.064 | -0.53 | .600 | -0.08 |
|  | **MSF** | **0.059** | **0.077** | **-3.14** | **.002** | **-0.44** |
|  | LSF | 0.880 | 0.858 | 1.99 | .048 | 0.28 |
|  | self-similarity | 0.60 | 0.58 | 1.16 | .245 | 0.17 |
|  | **complexity** | **35.94** | **30.16** | **4.91** | **< .001** | **0.70** |
|  | **anisotropy** | **1.07*10^-3^** | **1.20*10^-3^** | **5.32** | **<.001** | **-0.75** |
|  | hue | NA | NA | NA | NA | NA |
|  | saturation | NA | NA | NA | NA | NA |
|  | **brightness** | **0.69** | **0.74** | **-4.57** | **< .001** | **-0.65** |
|  | SD of hue | NA | NA | NA | NA | NA |
|  | SD of saturation | NA | NA | NA | NA | NA |
|  | **SD of brightness** | **0.45** | **0.43** | **3.17** | **.002** | **0.45** |
|  | **entropy** | **2.65** | **2.38** | **4.64** | **< .001** | **0.66** |
|  | **edge density** | **0.12** | **0.02** | **4.87** | **< .001** | **0.69** |
|  | **straight edge density** | **0.04** | **0.09** | **-14.90** | **< .001** | **-2.11** |
|  | **non-straight edge density** | **251.57** | **73.96** | **16.19** | **< .001** | **2.29** |
|  | **fractal dimension** | **1.80** | **1.76** | **4.07** | **< .001** | **0.58** |

Table S7: Mean values of image measures for the image set used in Study 2 (*n* = 20 per image category). Results of *t*-test (df=38) comparing nature and urban images. Rows in bold indicate significant difference between nature and urban images (critical *p*-value based on Bonferroni-correction: *p* = .0026).

| image type | image property | image category | | results of *t*-test | | |
| --- | --- | --- | --- | --- | --- | --- |
|  |  | nature | urban | *t* | *p* | *d* |
| original | **spectral slope** | **-2.22** | **-2.49** | **5.68** | **< .001** | **1.80** |
|  | **weighted residuals** | **3.38*10^12^** | **6.85*10^12^** | **-6.15** | **< .001** | **-1.94** |
|  | HSF | 0.011 | 0.011 | -0.07 | 948 | -0.02 |
|  | MSF | 0.029 | 0.037 | -0.74 | .462 | -0.24 |
|  | LSF | 0.960 | 0.952 | 0.58 | .595 | 0.17 |
|  | self-similarity | 0.61 | 0.60 | 0.24 | .814 | 0.08 |
|  | complexity | 12.03 | 11.40 | 0.57 | .568 | 0.18 |
|  | **anisotropy** | **4.10*10^-4^** | **6.68*10^-4^** | **-7.72** | **< .001** | **-2.44** |
|  | **hue** | **1.74** | **0.01** | **3.93** | **< .001** | **1.24** |
|  | **saturation** | **0.50** | **0.19** | **7.46** | **< .001** | **2.36** |
|  | brightness | 0.60 | 0.58 | 0.46 | .652 | 0.14 |
|  | SD of hue | 1.22 | 1.65 | -3.16 | .003 | -1.00 |
|  | **SD of saturation** | **0.25** | **0.17** | **4.32** | **< .001** | **1.37** |
|  | SD of brightness | 0.25 | 0.26 | -0.88 | .386 | -0.28 |
|  | entropy | 7.49 | 7.53 | -0.53 | .597 | -0.17 |
|  | **edge density** | **0.13** | **0.10** | **5.75** | **< .001** | **1.82** |
|  | **straight edge density** | **0.06** | **0.13** | **-6.51** | **< .001** | **-2.06** |
|  | **non-straight edge density** | **280.15** | **41.80** | **11.94** | **< .001** | **3.78** |
|  | fractal dimension | 1.74 | 1.68 | 2.30 | .027 | 0.73 |
| phase-scrambled | **spectral slope** | **-2.25** | **-2.49** | **4.55** | **< .001** | **1.44** |
|  | **weighted residuals** | **2.96*10^12^** | **6.06*10^12^** | **-5.87** | **< .001** | **-1.86** |
|  | HSF | 0.010 | 0.011 | -0.17 | .866 | -0.05 |
|  | MSF | 0.028 | 0.037 | -0.84 | .407 | -0.27 |
|  | LSF | 0.962 | 0.953 | 0.64 | .527 | 0.20 |
|  | self-similarity | 0.92 | 0.918 | -0.11 | .915 | -0.03 |
|  | complexity | 14.74 | 16.33 | -1.36 | .181 | -0.43 |
|  | anisotropy | 1.56*10^-4^ | 1.69*10^-4^ | -0.66 | .516 | -0.21 |
|  | **hue** | **1.60** | **-0.12** | **3.69** | **< .001** | **1.17** |
|  | **saturation** | **0.43** | **0.18** | **6.79** | **< .001** | **2.15** |
|  | brightness | 0.57 | 0.57 | 0.20 | .842 | 0.06 |
|  | **SD of hue** | **0.92** | **1.47** | **-5.17** | **< .001** | **1.63** |
|  | **SD of saturation** | **0.25** | **0.19** | **4.07** | **< .001** | **1.29** |
|  | SD of brightness | 0.23 | 0.24 | -1.08 | .289 | -0.34 |
|  | entropy | 7.71 | 7.79 | -1.70 | .097 | -0.54 |
|  | edge density | 0.17 | 0.16 | 3.07 | .004 | 0.97 |
|  | straight edge density | 0.05 | 0.06 | -1.38 | .177 | -0.44 |
|  | **non-straight edge density** | **480.45** | **404.00** | **4.46** | **< .001** | **1.41** |
|  | fractal dimension | 1.96 | 1.95 | 1.96 | .057 | 0.62 |

Table S8. Spearman correlations for image properties with restorativeness ratings of original images (*n* = 100 per image category). Rows in bold indicate significant correlations (critical *p*-value based on Bonferroni-correction: *p* = .0026).

| image property | *r_s_* | *p*-value |
| --- | --- | --- |
| **spectral slope** | **.37** | **< .001** |
| **weighted residuals** | **-.60** | **< .001** |
| **HSF** | **-.35** | **< .001** |
| **MSF** | **-.33** | **< .001** |
| **LSF** | **.34** | **< .001** |
| self-similarity | .06 | .363 |
| complexity | -.04 | .613 |
| **anisotropy** | **-.56** | **< .001** |
| **hue** | **.40** | **< .001** |
| **saturation** | **.55** | **< .001** |
| brightness | -.12 | .099 |
| **SD of hue** | **-.23** | **.001** |
| **SD of saturation** | **.39** | **< .001** |
| SD of brightness | -.05 | .488 |
| entropy | .21 | .003 |
| **edge density** | **.53** | **< .001** |
| **straight edge density** | **-.57** | **< .001** |
| **non-straight edge density** | **.72** | **< .001** |
| fractal dimension | .21 | .003 |

Figure S9. Histogram of sum score of the connectedness to nature questionnaire for the sample of Study 2.


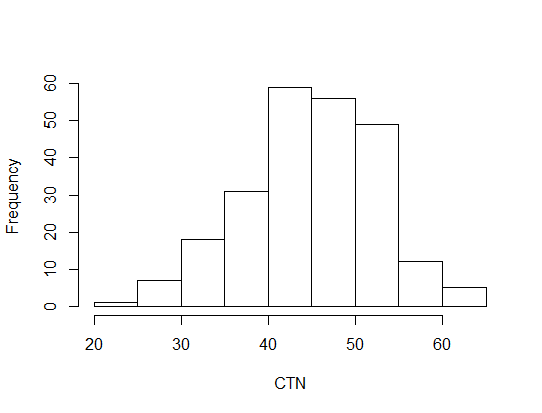


Table S10. Words (in German) used as attributes in Study 2.

| valence | | mood | | restoration/stress | |
| --- | --- | --- | --- | --- | --- |
| good | bad | positive | negative | restoration | stress |
| peace (Frieden) | war (Krieg) | happy (glücklich) | depressed (depressiv) | relaxation (Entspannung) | time pressure (Zeitdruck) |
| joy (Freude) | violence (Gewalt) | cheerful (fröhlich) | unhappy (unglücklich) | well-being (Wohlbefinden) | pressure (Druck) |
| love (Liebe) | abuse (Missbrauch) | joyful (freudig) | hopless (hoffnungslos) | vacation (Urlaub) | burden (Belastung) |
| freedom (Freiheit) | spitefulness (Gehässigkeit) | excited (begeistert) | desperate (verzweifelt) | calmness (Ruhe) | hectic rush (Hektik) |
| health (Gesundheit) | despair (Verzweiflung) | satisfied (zufrieden) | frustrated (frustriert) | balance (Ausgeglichenheit) | tenseness (Angespanntheit) |
| happiness (Glück) | hopelessness (Ausweglosigkeit) | buoyant (heiter) | sad (traurig) | break (Pause) | burnout (Burnout) |
| friend (Freund) | illness (Krankheit) | delighted (erfreut) | furious (zornig) | free time (Freizeit) | huffiness (Gereiztheit) |
| fun (Spaß) | poverty (Armut) | amused (vergnügt) | angry (wütend) | lightheartedness (Unbeschwertheit) | exhaustion (Erschöpfung) |
| hope (Hoffnung) | hell (Hölle) | in love (verliebt) | displeased (verärgert) | serenity (Gelassenheit) | compulsion (Zwang) |
| tenderness (Zärtlichkeit) | poison (Gift) | balanced (ausgeglichen) | aggressive (aggressiv) | pleasure (Genuss) | restlessness (Unruhe) |
